# Androgen Receptor Variants Occur Frequently in Castration Resistant Prostate Cancer Metastases

**DOI:** 10.1371/journal.pone.0027970

**Published:** 2011-11-17

**Authors:** Xiaotun Zhang, Colm Morrissey, Shihua Sun, Melanie Ketchandji, Peter S. Nelson, Lawrence D. True, Funda Vakar-Lopez, Robert L. Vessella, Stephen R. Plymate

**Affiliations:** 1 Department of Urology, University of Washington, Seattle, Washington, United States of America; 2 Department of Medicine, University of Washington, Seattle, Washington, United States of America; 3 Fred Hutchinson Cancer Research Center, Seattle, Washington, United States of America; 4 Department of Pathology, University of Washington, Seattle, Washington, United States of America; 5 GRECC and Research Service, Department of Veterans Affairs Medical Center, Seattle, Washington, United States of America; Florida International University, United States of America

## Abstract

**Background:**

Although androgens are depleted in castration resistant prostate cancer (CRPC), metastases still express nuclear androgen receptor (AR) and androgen regulated genes. We recently reported that C-terminal truncated constitutively active AR splice variants contribute to CRPC development. Since specific antibodies detecting all C-terminal truncated AR variants are not available, our aim was to develop an approach to assess the prevalence and function of AR variants in prostate cancer (PCa).

**Methodology/Principal Findings:**

Using 2 antibodies against different regions of AR protein (N- or C-terminus), we successfully showed the existence of AR variant in the LuCaP 86.2 xenograft. To evaluate the prevalence of AR variants in human PCa tissue, we used this method on tissue microarrays including 50 primary PCa and 162 metastatic CRPC tissues. RT-PCR was used to confirm AR variants. We observed a significant decrease in nuclear C-terminal AR staining in CRPC but no difference between N- and C-terminal AR nuclear staining in primary PCa. The expression of the AR regulated proteins PSA and PSMA were marginally affected by the decrease in C-terminal staining in CRPC samples. These data suggest that there is an increase in the prevalence of AR variants in CRPC based on our ability to differentiate nuclear AR expression using N- and C-terminal AR antibodies. These findings were validated using RT-PCR. Importantly, the loss of C-terminal immunoreactivity and the identification of AR variants were different depending on the site of metastasis in the same patient.

**Conclusions:**

We successfully developed a novel immunohistochemical approach which was used to ascertain the prevalence of AR variants in a large number of primary PCa and metastatic CRPC. Our results showed a snapshot of overall high frequency of C-terminal truncated AR splice variants and site specific AR loss in CRPC, which could have utility in stratifying patients for AR targeted therapeutics.

## Introduction

Metastatic prostate cancer (PCa) that recurs following castration or androgen deprivation therapy (ADT), termed castration resistant prostate cancer (CRPC), portends a poor outcome with high lethality. Although circulating levels of androgens are depleted in CRPC, tumor progression is often concomitant with elevated levels of the androgen receptor (AR), activation of the AR, and the expression of AR-regulated genes. However, an increase in AR expression by itself is generally not sufficient to engage the AR transcriptional program [1]. Various mechanisms have been shown to lead to AR transactivation and engage the AR program following castration. These include persistence of intratumoral androgens, ectopic androgen synthesis by the tumor either from adrenal androgens or intratumoral *de novo* synthesis, and enhanced androgen transport into the tumor by solute carrier organic anion transporter proteins [2]–[6]. Several cytokines and growth factor pathways have been shown to be able to activate the AR through direct binding or cross-talk mechanisms [7]–[13]. Alterations in AR co-regulators may also modulate AR activity when androgen levels are decreased [14]–[18]. Functionally, each of these mechanisms promoting AR activation in CRPC requires the carboxy-terminus region of the mature protein which contains the ligand-binding domain (LBD).

In addition to mechanisms leading to AR activation in CRPC that require ligand, recent evidence points to the existence of alternatively spliced forms of AR mRNAs that encode receptors devoid of the LBD, but retaining the ability to engage transcriptional machinery and promote the regulation of known---and potentially new---sets of transcriptional targets [19]–[26]. Not only are these C-terminal truncated AR variants constitutively active, but their structure predicts a general resistance to therapeutics such as AR-antagonists that require binding to the LBD for activity. To date, we and others have identified three AR splice variants in human tissue specimens [22], [25]–[27]. AR-V1 encodes a splice variant comprised of exons 1–3 and ending in a cryptic exon (CE1), AR-V7 (also named AR3) encodes a protein with exons 1–3 and a terminal cryptic exon (CE3), and AR^v567es^ encodes a protein comprised of exons 1–4, and because of a frame-shift due to loss of exons 5–7, exon 8 has a stop codon generated after the first 10 amino acids resulting in a shortened exon8. [22], [25]–[27]. Additional AR splice variants have been detected in human PCa cell lines [21], [24], [26]–[28].

Several studies evaluating the expression of AR splice forms in a small number of prostate cancers suggest that AR variants are more readily detected in CRPC compared to hormone-naïve cancers, and may emerge due to the selective pressure of AR targeted therapy [22], [25], [26]. A recent study used qRT-PCR to identify AR variant transcripts in 40 bone metastasis, of which 30 were from CRPC, and found an association between the expression of AR variants and survival [29]. Determining the prevalence of AR variants in different clinical states of prostate cancer has been challenged by requirements for well-preserved frozen tissue samples for transcript-based analyses, and the lack of antibodies capable of specifically detecting most AR variant proteins. To overcome this limitation, we sought to take advantage of the fact that new AR carboxy-termini encoded by alternatively spliced forms of the AR mRNA cannot be recognized by antibodies directed against the normal C-terminus of the full-length AR (AR^FL^). We hypothesized that this feature of AR variants afforded an opportunity to identify AR protein variants in formalin-fixed tissues by comparing the differential staining of antibodies recognizing either N- or C-terminus of the AR. Therefore, in the present study, we used antibodies against the N- or C-terminus of the AR protein to interrogate a large number of benign prostate tissue, primary hormone naïve PCa and a series of metastatic CRPC to ascertain the prevalence of C-terminal truncated AR variants.

## Materials and Methods

### Reagents

The antibodies used in this study and the working conditions are listed in Table 1.

**Table 1:** Antibodies used in this study.

Antibody	Company	Clone/lot	IHC Dilution	WB Dilution
Androgen Receptor	Biogenex	F39.4.1	1∶60	
Androgen Receptor	Santa Cruz	441		1∶4000
Androgen Receptor	Santa Cruz	C-19	1∶200	1∶2000
Chromogranin A	Dako	DAK-A3	1∶100	
Synaptophysin	Santa Cruz	D-4	1∶200	
PSA	Dako	A0562	1∶200	
PSMA	Invitrogen	18-7318	1∶35	
Ki67	Dako	MIB-1	1∶100	
AKT-1	Calbiochem	Ab-1	1∶2000	
Mouse IgG	Abcam	MOPC-21		
Rabbit IgG	Vector laboratories	S0818		

### Tissue

Human primary and metastatic PCa tissues were obtained as part of the PCa research program and University of Washington Medical Center Prostate Cancer Donor Rapid Autopsy Program, which is approved by the University of Washington Institutional Review Board. The Institutional Review Board of the University of Washington Medical Center approved all procedures involving human subjects, and all subjects signed written informed consent. Human tissue microarrays (TMAs) consists of 42 patients from Prostate Cancer Donor Rapid Autopsy Program (including 65 soft tissue metastases and 120 bone metastases) [30], 55 radical prostatectomy patients (including 28 normal prostate, 24 hyperplastic prostate, and 50 primary prostate cancer tissues) were used. The LuCaP 86.2 prostate cancer xenograft is an adenocarcinoma that does not respond to castration and was derived from a human PCa bladder metastasis. The LuCaP 35 prostate cancer xenograft is an adenocarcinoma that responds to castration and was derived from a PCa lymph node metastasis. These xenografts fail to grow as cell lines, thus they are maintained by serial passage in SCID mice. The LuCaP 86.2 xenograft expresses a known C-terminal truncated AR variant AR^v567es^ that is constitutively active. The LuCaP 35 xenograft only expresses wide type AR [25]. Fresh LuCaP 35 and 86.2 xenograft tissues were used for western analysis. Twenty two CRPC metastatic tissues from rapid autopsy patients corresponding to the tissues on the human TMA had been snap frozen in liquid nitrogen after resection and stored at -80°C until use. Clinical data relating to the 42 autopsy patients is shown in Table 2.

**Table 2:** Clinical data of 42 CRPC patients*.

	Min-max values	Mean	Average
Age at PCa Diagnosis	42–93	63	64
Diagnosis to Death (years)	0–20	5	6.5
Castration to Death (month)	1–92	19.5	30
PSA at Diagnosis (ng/ml)	1.7–4000	12.4	267.22
Final PSA (ng/ml)	0.15–7402	413.2	843.48

*All 42 patients had castrate resistant prostate cancer at the time of autopsy, defined by the presence of a rising serum PSA following medical or surgical castration. All patients' tissues were obtained at autopsy under University of Washington Medical Center Prostate Cancer Donor Rapid Autopsy Program.

### Reverse Transcription-PCR (RT-PCR) and quantitative RT-PCR (qRT-PCR)

Total tissue RNA was isolated from minced fresh tissue using Trizol reagent (Invitrogen) according to the manufacturer's instructions. Two micrograms of total RNA was digested with DNase I, and reverse transcribed using Superscript First-Strand Synthesis System (Invitrogen). PCR was performed using AmpliTaq Gold® PCR Master Mix (Applied Biosystems). PCR products were run on 2% agarose gel and image pictures were taken by using AlphaDigiDoc Pro imaging system from Alpha Innotech (San Leandro, CA). qRT-PCR reactions were done using an Applied Biosystems 7900 sequence detector with 5 ng of cDNA, 200 nM of each primer pair and Power SYBR Green PCR Master Mix or TaqMan Universal PCR Master Mix from Applied Biosystems.

Gene expression levels were measured by relative quantification between RNA samples, and fold expression changes were determined by the 2–ΔΔCT method [31]. All qRT-PCR experiments were performed in triplicate, and the housekeeping gene RPL13A was used as an endogenous control.

The primer sequences are listed in Table 3.

**Table 3:** Primer sequences.

Target gene	Primer Sequence (5′ to 3′)
AR^FL^	F: ACATCAAGGAACTCGATCGT ATCATTGC
	R: TTGGGCACTTGCACAGAGAT
AR^v567es^	F: TGCTGGACACGACA ACAA
	R: GCAGCTCTCTCGCAATCA
AR-V7	F: CCATCTTGTCG
	TCTTCGGAAATGTTATGAAGC
	R: TTTGAATGAGGCAAGTCAGCCTTT CT
AR3	F: CTACTCCGGACCTTACGGGGACATGC G
	R: TGCCAACCCGGAATTTTTCTCCC
RPL13A	F:CCTGGAGGAGAAGAGGAAAGAGA
	R:TTGAGGACCTCTGTGTATTTGTCAA
CDK1	F: GGAAACCAGGAAGCCTAGCATC
	R: GGATGATTCAGTGCCATTTTGCC
CYCLINA2	F: CTCTACACAGTCACGGGACAAAG
	R: CTGTGGTGCTTTGAGGTAGGTC
C-MYC	F: CCTGGTGCTCCATGAGGAGAC
	R: CAGACTCTGACCTTTTGCCAGG
UGT2B17	F: ACCAGCCAAACCCTTGCCTAAG
	R:GGCTGATGCAATCATGTTGGCAC
CDC20	F: CGGAAGACCTGCCGTTACATTC
	R: CAGAGCTTGCACTCCACAGGTA
AKT1	F: TGGACTACCTGCACTCGGAGAA
	R: GTGCCGCAAAAGGTCTTCATGG
UBE2C	F: TGGTCTGCCCTGTATGATGT
	R: AAAAGCTGTGGGGTTTTTCC

### Cell Culture and stimulation

VCAP cells that expressed both AR^FL^ and AR^v567es^ variant were grown to 80% confluence in 30 mm plates in RPMI 1640 medium with 5% serum, and then switched to RPMI-1640 medium with 5% charcoal stripped serum for 24 h. Dihydrotestosterone (DHT) 10^−9^ M, MDV-3100 50 nM, or MDV-3100 plus DHT were added to the cultures. After 24 h, total RNA was collected from duplicate wells for qRT-PCR to detecting AR^FL^, AR^v567es^ and AR-V7 transcripts. The experiment was repeated 6 times with triplicates each time, and the qRT-PCR results were normalized to DHT treatment group.

### Western Blotting

Fresh tissue was homogenized and lysed with cold lysis buffer (50 mM HEPES, 150 mM NaCl, 1.5 mM MgCl_2_, 1 mM EGTA, 1% Triton X-100) containing Halt™ Phosphatase Inhibitor Cocktail and protease inhibitors (Thermoscientific). Complete lysates were separated on SDS-PAGE, transferred onto a nitrocellulose membrane, blocked in 5% milk-PBS-Tween and probed with respective overnight at 4°C. Membranes were incubated with a horseradish peroxidase-conjugated secondary antibody (Cell Signaling), and developed with ECL (Pharmacia Biotech). The membranes were stripped for 30 min in Stripping Buffer (Thermoscientific) and re-probed with anti-β-actin antibody as a loading control (Sigma-Aldrich). Independent experiments validated that this stripping procedure did not lead to loss of signal.

### Immunohistochemistry

Formalin-fixed paraffin-embedded tissue sections (5μm) were deparaffinized and rehydrated. Antigen retrieval was performed with 10 mM citrate buffer (pH 6.0) in a pressure cooker (20 psi for 10 min). Endogenous peroxide and biotin/avidin was blocked for 15 min with respective agents (Vector Laboratories). After incubating with 5% normal goat-horse-chicken serum at room temperature for 1 h, sections were incubated with primary antibodies (table 1) at 4°C overnight followed by biotinylated secondary antibodies and sABC reagent (Vector Laboratories). DAB (Invitrogen) was used as the chromogen, and hematoxylin as counterstain. Mouse or Rabbit IgG, as appropriate, at the same concentration as the primary antibody was used as a negative control and did not show nonspecific staining [32].

### Immunohistochemical assessment

A few unusable cores were found in the TMAs due to tissue core missing, cancer necrosis, or insufficient cancer cells. These cores were excluded from the results.

Immunostaining was assessed using a quasi-continuous nuclear AR score, created by multiplying each intensity level (0 for no stain, 1 for weak stain, and 2 for intense stain) by the corresponding percentage of positive cells, and then summing the results.

Ki-67 staining was measured by randomly choosing up to 4 fields of 250 µm^2^ in each tissue site. Total cell number and Ki67 positive cell number were counted, and the final Ki-67 index was calculated using positive cell number divided by total cell number.

AR antibodies used in this study targeted three distinct regions of the human AR protein. The immunogens of AR F39.4.1 (against aa301–320) and AR441 (against aa299–315) were located in the N-terminal of AR while AR C-19 (against aa900–919) in the C-terminal. Therefore, we describe AR F39.4.1 and AR441 as N-terminal AR antibodies and AR C-19 as a C-terminal AR antibody. Specific patterns of AR nuclear immunostaining were assessed as: N+C+ (similar positive N-terminal and C-terminal AR staining in nucleus); N+C↓ (C-terminal AR score dropped more than 50% compared to N-terminal in nucleus) and N-C- (similar negative N- and C-terminal AR staining in nucleus). The tissues in N↓C+ group were not included because they were rare, and not relative to C-terminal truncated AR variants.

### Statistical analysis

Statistical analyses of the results were performed using Prism software (Prism Graphpad), where we used the Mann-Whitney test, and *p* values ≤0.05 were chosen as statistical significant.

## Results

### Alternatively spliced forms of AR could be identified using N- and C-terminal antibodies

Several AR transcript variants have been described that encode AR polypeptides devoid of the C-terminal LBD due to alternative exon splicing [21], [24]–[28]. However, specific antibodies are not available to detect all of these variants in tissue. As antibodies have been developed toward specific N-terminal and C-terminal domains of AR protein, we sought to determine if these reagents could be used to distinguish PCa expressing different AR forms. To validate this approach, we first evaluated two PCa xenograft lines derived in the laboratory of one of the authors (RLV) and known to express different AR-encoding mRNAs. The LuCaP 86.2 xenograft, derived from a human PCa bladder metastasis and maintained by serial passage in uncastrated SCID mice, has been shown to possess a C-terminal truncated AR splice variant that skipped exons 5–7 and encodes an alternate reading frame from exon 8, designated AR^v567es^. The LuCaP 35 xenograft was derived from a PCa lymph node metastasis and expresses full-length AR (AR^FL^)-encoding mRNA comprised of 8 exons and a protein of appropriate size for this transcript [25].

**Figure 1:**
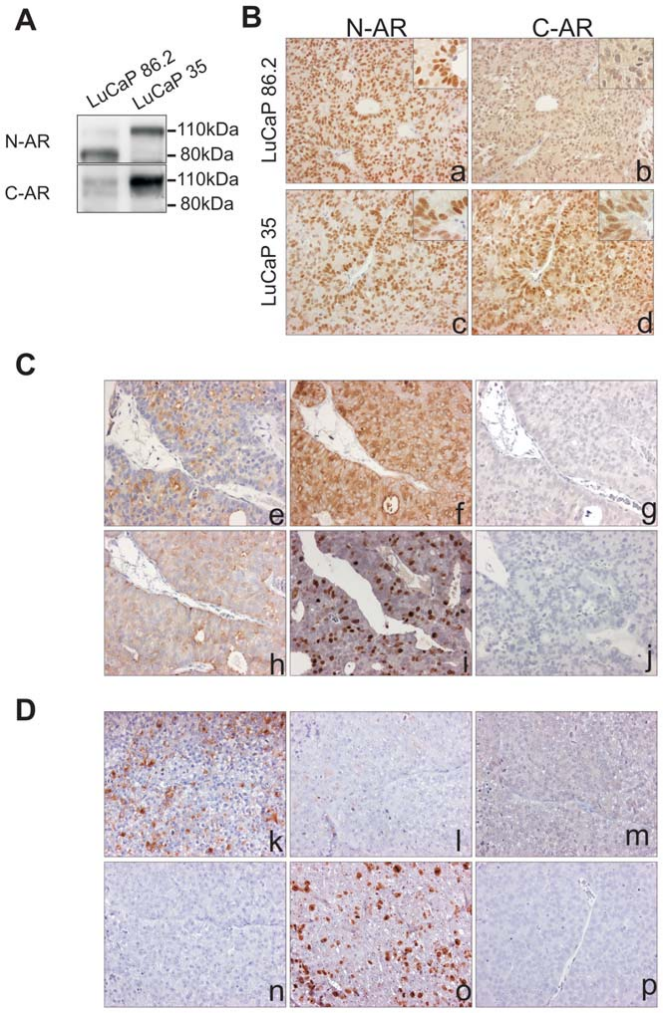
Characterization of the LuCaP 86.2 subcutaneous xenograft tumor. (**A**) Western blot analysis of AR expression in LuCaP 86.2 and LuCaP 35 xenograft tumors. (**B**) IHC staining for the N- and C-terminal AR on LuCaP 86.2 (a and b) and LuCaP 35 (c and d) xenograft tumors. (**C**) IHC staining for PSA (e), PSMA (f), Chromogranin-A (g), Synaptophysin (h), Ki67 (i) and negative control (j) on LuCaP 86.2 xenograft. (**D**) IHC staining for PSA (k), PSMA (l), Chromogranin-A (m), Synaptophysin (n), Ki67 (o) and negative control (p) on LuCaP 35 xenograft. (original magnification x200, insert x400).

We analyzed proteins derived from LuCaP 86.2 and LuCaP 35 xenografts by Western blot using antibodies recognizing N-terminus (AR 441) or C-terminus (AR C-19) of AR^FL^ protein. In the LuCaP 35 tumor, both AR antibodies detected a similar 110 KDa AR polypeptide that corresponded to the size of AR^FL^. In the LuCaP 86.2 tumor, beside a weak 110 KDa band, N-terminal AR antibody also identified an 80 KDa AR isoform that corresponded to the predicted size of the C-terminal truncated AR splice variant-AR^v567es^ while C-terminal AR antibody did not (Figure 1A).

Similar to the Western blot result, immunohistochemical analysis (IHC) of LuCaP 86.2 showed very weak nuclear staining using the C-terminal AR (AR c-19) antibody, but intense nuclear staining in the serial section with AR antibody against N-terminal (AR F39.4.1). The LuCaP 35 tumor, shown to express only AR^FL^ by Western analysis, did not show any difference between N- and C-terminal AR staining by IHC (Figure 1B). These data confirmed that comparing the N-terminal AR with C-terminal AR expression could identify C-terminal truncated AR variants/mutations in PCa tissue.

AR^v567es^ has been shown to be constitutively active [25], this is consistent with the fact that different AR immunoreactivity with N- and C-terminal antibodies was observed in the nucleus. To further confirm nuclear AR activity, we stained for the AR-regulated PSA and PSMA proteins. LuCaP 86.2 was immunoreactive for both PSA and PSMA. To confirm that LuCaP 86.2 was not a neuroendocrine xenograft line, we stained with neuroendocrine biomarkers Chromogranin A (CHG-A) and Synaptophysin (SYN). LuCaP 86.2 showed negative CHG-A immunoreactivity, and weak to moderate SYN immunoreactivity as a fine, granular reaction product that was predominantly localized in the peripheral cytoplasm of cells. Furthermore, approximately 20% of the tumor cells were Ki67 positive indicating LuCaP 86.2 had a moderate proliferation rate (Figure 1C). Corresponding IHC results for LuCaP 35 are also shown in Figure 1D.

### Variations in N-terminal and C-terminal AR expression occurred rarely in benign epithelium and primary untreated PCa

**Figure 2:**
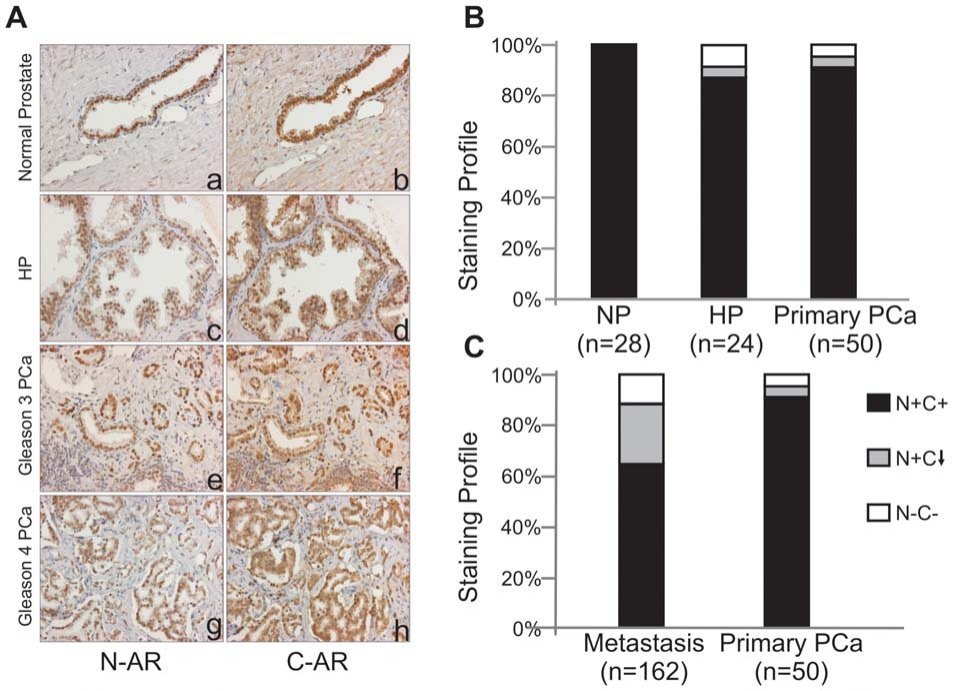
AR staining profiles of normal prostate, primary PCa and CRPC. (**A**) IHC staining for N- and C-terminal AR in normal prostate (NP) (a and b), hyperplastic prostate (HP) (c and d) and primary PCa (e-h) (magnification x200). (**B**) Comparison of AR staining profiles among normal prostate, hyperplastic prostate and primary PCa. (**C**) Comparison of AR staining profiles between primary PCa and metastatic CRPC.

To determine the frequency of C-terminal truncated AR variants in benign epithelium and untreated localized PCa, we scored IHC staining of radical prostatectomy specimens. All 28 normal prostate specimens had concordant N-terminal and C-terminal nuclear AR expression (N+C+) (Figure 2A a and b). Among the 24 hyperplastic prostate samples, 21 cases (87.5%) showed consistent N+C+ expression (Figure 2A c and d), only 1 (4.2%) had decreased C-terminal AR staining (N+C↓) and 2 (8.3%) did not have any AR immunoreactivity (N-C-). In 50 primary PCa samples, 46 cases (92%) expressed N+C+ AR (Figure 2A e–h), 2 cases (4%) had decreased nuclear C-terminal vs. N-terminal AR staining (N+C↓), while 2 cases (4%) were AR negative (N-C-). Overall, there was no significant difference in the ratio of N- vs. C-terminal nuclear staining intensity between normal prostate and hyperplastic prostate samples (*p* = 0.5756), normal prostate and PCa samples (*p* = 0.7428), and hyperplastic prostate and PCa samples (*p* = 0.7508) (Figure 2B).

### Variations in N-terminal and C-terminal AR expression occurred frequently in CRPC

To determine the frequency of variant AR expression in metastatic PCa, we scored IHC staining of metastatic sites from 42 patients who died of advanced CRPC.

Among 162 metastatic sites, 103 (63.6%) sites showed consistent nuclear AR expression with both antibodies (N+C+). However, 39 (24.1%) sites had a nuclear C-terminal AR score (N+C↓) that was at least 50% less than the corresponding N-terminal AR score, and 20 (12.3%) sites of metastasis had no nuclear AR expression (N-C-) (Figure 2C). These IHC results were in agreement with the frequency of AR splice variants in metastatic PCa determined using PCR methods to detect AR-V7/AR3 and AR^v567es^ transcripts [25]. There was no difference in N-terminal AR staining between primary and metastatic PCa (*p* = 0.38, data not show), but the frequency of decreased nuclear C-terminal AR expression in metastatic CRPC was significantly higher than in primary PCa (*p* = 0.0027). The comparison of nuclear AR expression between primary PCa and metastatic CRPC is shown in Figure 2C.

### AR-regulated gene expression was altered in metastatic CRPC with diminished nuclear C-terminal AR

**Figure 3:**
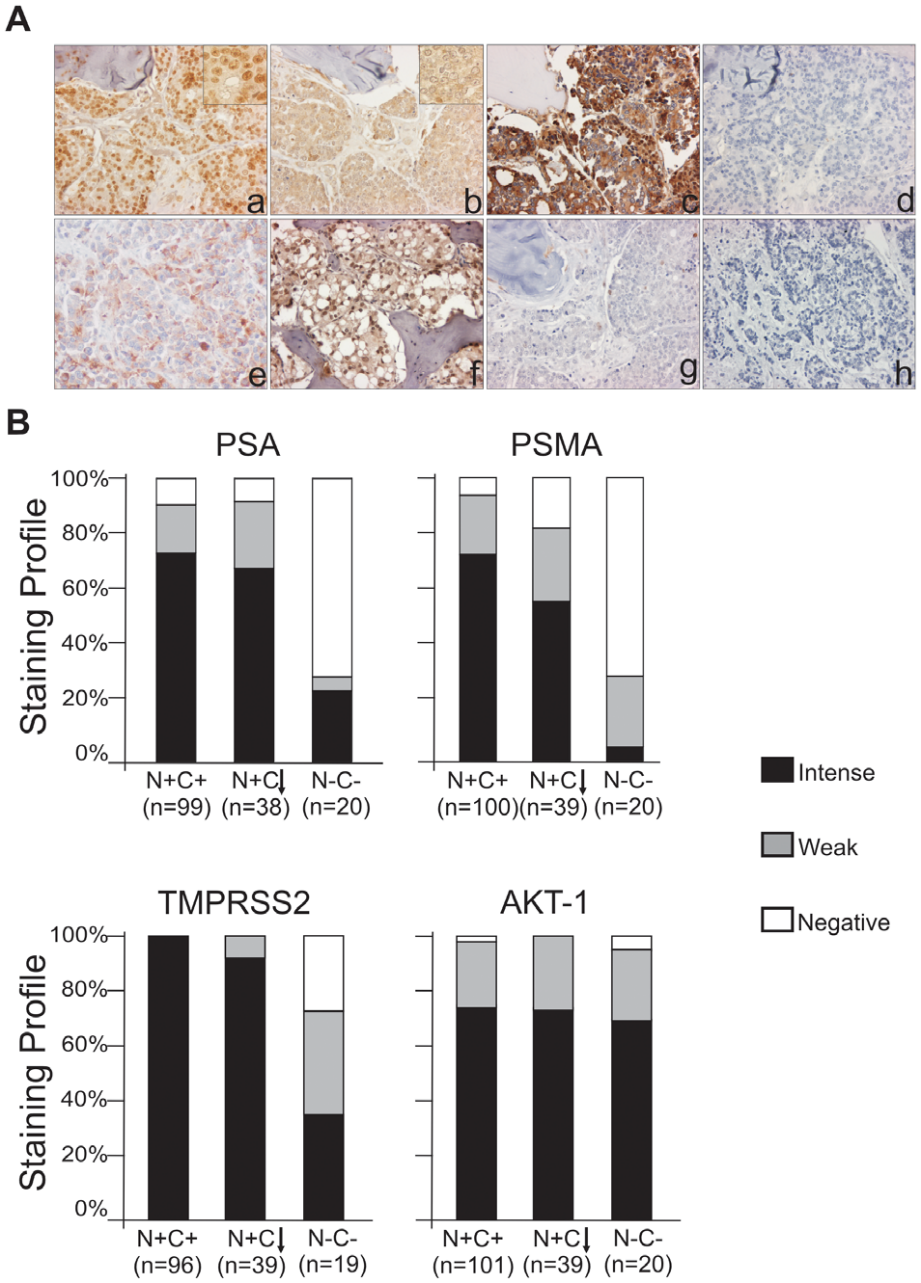
Expression of AR variants and AR regulated proteins in metastatic CRPC. (**A**) IHC staining for N-terminal AR (a), C-terminal AR (b), PSA (c), PSMA (d), TMPRSS2 (e), AKT-1 (f), Ki-67(g), Negative control (h) on a metastatic CRPC tissue (magnification x200, insert x400). (**B**) PSA, PSMA, TMPRSS2 and AKT-1 staining profiles of CRPC.

To determine if the decrease in nuclear C-terminal AR expression was associated with an alteration in the expression of AR regulated proteins, representing a loss of AR activity, we examined AR regulated proteins PSA, PSMA, and TMPRSS2 expression in the metastatic CRPC samples (Figure 3A). While barely missing significance, the expression of PSA in N+C↓ metastatic sites was lower than N+C+ metastatic sites (*p* = 0.0505). PSA expression in N-C- metastatic sites was significantly lower than both N+C+ and N+C↓ metastatic sites (*p*<0.001) (Figure 3B). Additionally, the expression of PSMA in N+C+ metastatic sites was higher than in N+C↓ metastatic sites (*p* = 0.0097), but PSMA in N-C- metastatic sites was significantly lower than both N+C+ and N+C↓ metastatic sites (*p*<0.0001) (Figure 3B). Finally, the expression of TMPRSS2 in N+C+ metastatic sites was significantly higher than N+C↓ metastatic sites (*p* = 0.045). TMPRSS2 in N-C- metastatic sites was significantly lower than both N+C+ and N+C↓ metastatic sites (*p*<0.01) (Figure 3B). The loss of TMPRSS2 expression in N+C↓ and N-C- metastatic sites was not as pronounced as the loss of PSA and PSMA expression, suggesting that TMPRSS2 may not be regulated by the AR at the same level as PSA and PSMA in CRPC.

**Figure 4:**
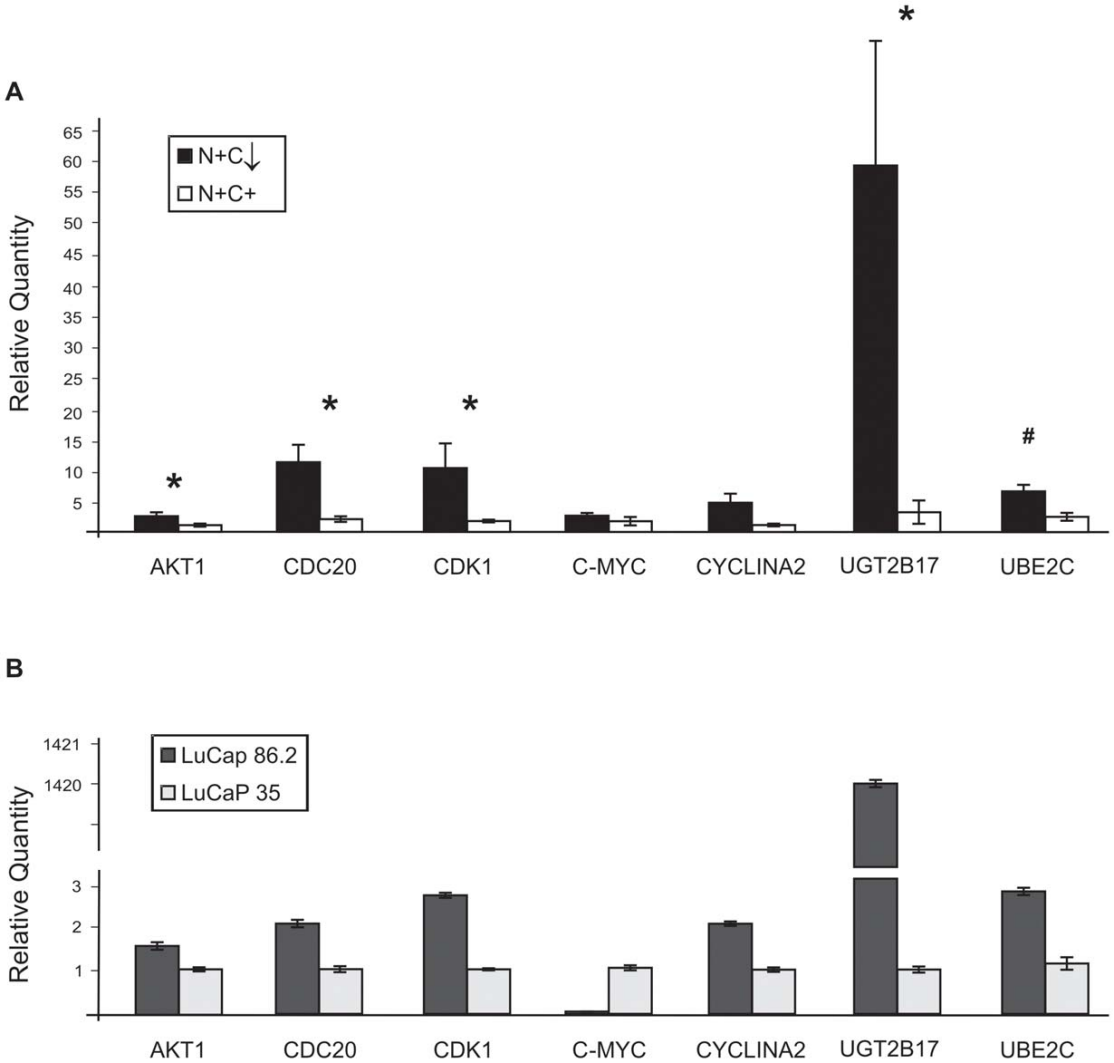
Quantitative RT-PCR of genes associated with AR C-terminal truncated variants. (**A**) Profile of seven AR variant associated genes expression in human metastatic tissues. (**B**) The same genes were measured in LuCaP 86.2 and LuCaP 35 xenografts. The housekeeping gene RPL13A was used as an endogenous control.

We next examined the genes that have been identified as having their expression increased in response to AR C-terminus truncated variants [26], [29], including AKT1, CDC20, CDK1, C-MYC, CyclinA2, UGT2B17 and UBE2C. Quantitative RT-PCR results showed that AKT1, CDC20, CDK1 and UGT2B17 expression were significantly higher in the N+C↓ (n = 11) compared to N+C+ samples (n = 7), (p< 0.05) (Figure 4A). UBE2C also trended higher but did not reach significance (p<0.10). We further detected AKT-1 protein expression by IHC. However, the relatively low cycle number indicated relatively high levels of gene expression in all cases. Therefore, when we also stained the TMA for AKT1, relatively strong signal was present in most of the tumor specimens on the TMA and given the semi quantitative measurements, no differences were detected between the groups by IHC (Figure 3B). Thus, cancers with N+C↓ AR expressed higher AKT1 levels, but this difference could not be detected on IHC due to abundant protein in all groups.

### The loss of nuclear C-terminal AR immunoreactivity was associated with the expression of AR variants in metastatic CRPC

We determined that loss of C-terminal AR immunoreactivity occurred frequently in metastatic CRPC. This loss of C-terminal immunoreactivity might be due to the expression of a number of known (e.g. AR-V7/AR3, or AR^v567es^) and unknown AR splice variants. To determine if the N- and C-terminal immunoreactivity was associated with AR transcript variants, we performed RT-PCR on a subset of metastatic CRPC tissues and compared the RT-PCR results to IHC results (Table 4). Of 4 N+C+ metastatic samples examined by RT-PCR, all expressed AR^FL^ (one also had limited expression of AR-V7). Of 8 metastatic samples classified as N+C↓ by IHC analysis, all expressed the AR^FL^ and 6 (75%) also expressed at least one known AR variant (AR-V7/AR3 and/or AR^v567es^) by RT-PCR. These N+C↓ metastatic samples also exhibited lesser levels of PSA and PSMA immunoreactivity. None of the four N-C- metastatic samples expressed the AR^FL^ examined by RT-PCR, although one showed very low level of AR^v567es^ expression.

**Table 4:** Comparison of IHC with RT-PCR results in PCa Metastases.

GAPDH	AR-V7/AR3	AR^v567es^	AR^FL^					
RT-PCR				Independent Metastatic Sites	Case Number	AR	PSA	PSMA
						IHC		
+	−	−	+	Lymph Node	04-050G	N+C+	+	+
+	−	−	+	Lymph Node	04-050R	N+C+	+	+
+	−	−	+	Lymph Node	04-112H	N+C+	−	±
+	±	−	+	Lymph Node	05-217F	N+C+	+	+
+	−	−	−	Liver	03-192A	N-C-	−	−
+	−	−	−	Lymph Node	03-192D	N-C-	−	−
+	−	±	−	Liver	05-144E	N-C-	−	−
+	−	−	−	Lymph Node	05-144H	N-C-	−	−
+	+	−	+	Liver	99-091C	N+C↓	±	+
+	+	−	+	Lymph Node	00-140J	N+C↓	±	+
+	−	−	+	Lymph Node	00-140N	N+C↓	±	±
+	+	+	+	Liver	05-187E	N+C↓	+	+
+	+	−	+	Liver	05-187F	N+C↓	+	+
+	±	−	+	Liver	05-011F	N+C↓	±	−
+	−	−	+	Lymph Node	05-214 I	N+C↓	+	−
+	+	−	+	Lymph Node	06-047H	N+C↓	+	+

+Intense expression, ±Limited/weak expression, − No expression.

These data demonstrated that IHC analysis using different AR antibodies recognizing distinct AR protein regions was highly concordant with transcripts encoding AR^FL^ and AR splice variants that lack C-terminal exons.

### AR variant mRNA was sensitive to androgen concentration in vitro

**Figure 5:**
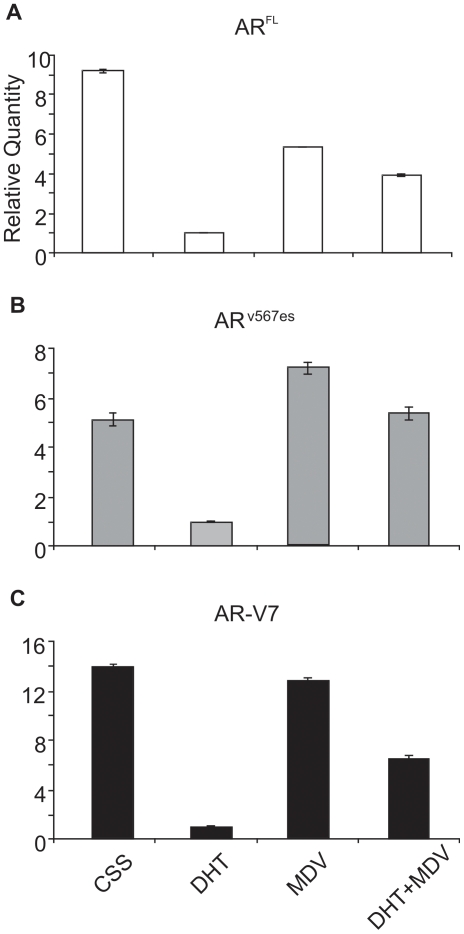
AR variant mRNA was sensitive to androgen concentration in vitro. (**A**) AR^FL^ mRNA was suppressed by DHT, MDV-3100 and MDV-3100+DHT but not to the level seen by DHT alone. (**B**) AR^v567es^ mRNA was suppressed in the presence of DHT, further increased by addition of MDV-3100 and suppressed to the level of CSS when DHT was added along with MDV-3100. (**C**) AR-V7 mRNA responded in a similar manner as AR^FL^.

VCAP cells were cultured in charcoal striped serum (CSS), and then treated with Dihydrotestosterone (DHT), MDV-3100 or MDV-3100 with DHT, separately. qRT-PCR showed that AR^FL^ mRNA was suppressed by adding DHT, MDV-3100 and DHT+MDV (Figure 5A). The variant AR^v567es^ mRNA was also suppressed by DHT; however, it could be increased by addition of androgen receptor antagonist MDV-3100 alone, and suppressed to the level of CSS when DHT was added with MDV-3100 (Figure 5B). AR-V7 mRNA responded in a similar manner as AR^FL^ (Figure 5C).

### Heterogeneous expression of AR was observed in individual patients

**Figure 6:**
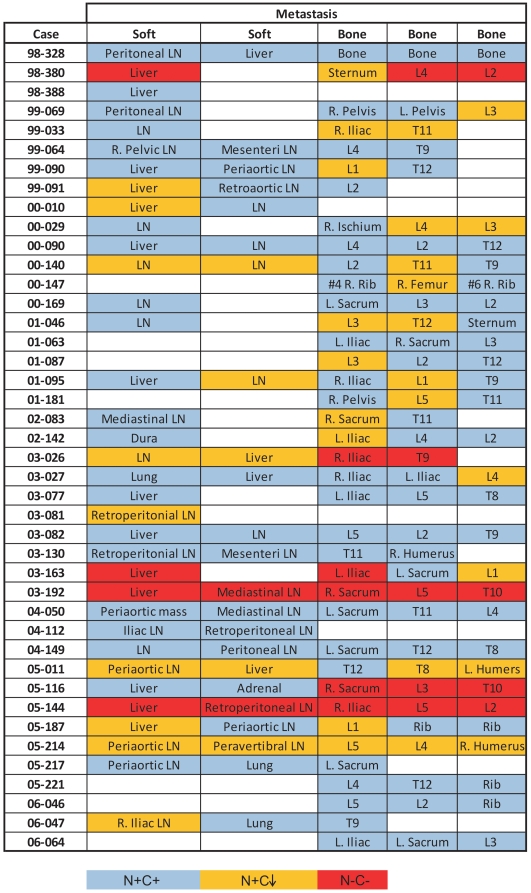
The heterogeneity of AR expression in individual patients. Multiple metastatic sites of 42 CRPC patients had been analyzed by IHC using 2 AR antibodies. The staining results were summarized as N+C+ (blue), N+C↓ (orange) and N-C- (red). LN = lymph node; L =  lumbar vertebra; R. =  right; L. =  left; T =  thoracic vertebra.

We identified C-terminal truncated AR proteins in multiple metastatic sites from each of 42 CRPC patients (Figure 6). Of the 42 patients, 16 (38%) had no loss of C-terminal AR expression, 23 (55%) had at least one metastatic site with decreased nuclear C-terminal AR immunoreactivity, and 6 (14.3%) had at least one site with no AR expression. These data highlight the heterogeneity of AR expression among different metastatic sites within the same patient.

Additionally, to determine if AR status promoted the rate of tumor growth, we divided the CRPC bone metastasis samples into three groups: N+C+ (n = 93), N+C↓ (n = 39), and N-C- (n = 18). The Ki67 index for the N+C+ sites was 18%. This was not significantly different from the N+C↓ sites (20.8%) (p = 0.4225) or the N-C- sites (17.8%) (p = 0.95).

## Discussion

Traditional concepts of AR translocation to the nucleus that involve ligand binding, dissociation from chaperones and nuclear translocation imply that without androgen ligand, AR would be found primarily in the cytoplasm and CRPC tumor progression would be driven by mechanisms other than those involving AR. However, this is not the case excepting most neuroendocrine tumors. CRPC usually possess a transcriptionally active AR that modulates the expression of AR regulated messenger RNAs [1], [33]. Furthermore, in most studies on CRPC tissues, the AR has been found in the nucleus.

Multiple mechanisms have been proposed to explain AR nuclear localization and some or all may be responsible for that seen in CRPC [34], [35]. Most of the proposed mechanisms would be able to translocate the AR to the nucleus since they require ligand binding to the LBD. If this was the case, assuming that there are no differences in AR structures, we would expect to see no differences in AR immunostaining with N- or C-terminal directed antibodies in metastatic CRPC. However, as we show in this study, there is a significant difference between nuclear N- and C- terminal AR antibodies in the metastatic tissues. This suggests that the mechanism(s) other than the ones mentioned above could also be involved during AR translocation into the nucleus without ligand in CRPC.

Although antibodies have been developed for the AR-V7/AR3 and AR^v567es^ splice variants, at least 20 AR splice variants have been reported to date. With specific antibodies available for only 2 of the variants, the use of specific antibody staining on tissue to detect castration-induced C-terminal truncated AR variants is not feasible [20]–[25]. Here, we develop a novel and rapid immunohistochemical approach that compares N- and C-terminal AR immunoreactivity, which can successfully show the overall frequency of C-terminal truncated AR splice variants in patients.

The data reported here correlating the expression of AR protein by IHC with 2 antibodies and validation of the expression of AR splice variants by RT-PCR, strongly suggest that the variation between AR N- and C-terminus immunoreactivity results from the expression of alternative AR mRNAs. However, alternative explanations should be entertained. The primary tumors and soft tissue metastases were formalin fixed and paraffin embedded while the metastatic lesions of bone were formalin fixed and decalcified with 10% formic acid before embedding in paraffin. We observed no significant difference in AR staining in soft tissue versus bone metastasis (p>0.05, data not shown). Furthermore, we have not seen differences with other antibodies between bone and soft tissue preparation methods using the same tissues and methods [36], [37]. Therefore we have not been able to identify a technical reason for the differences in staining. Thus, we conclude that the observed differential staining is due to the presence of variant AR transcripts that lack part or all of the C-terminus.

As we have recently reported, these variants are associated with androgen deprivation therapy (ADT) and occur relatively rapidly if ADT results in a significant decrease in circulating and intratumoral androgens [25]. Both AR variant mRNA and protein are very sensitive to androgen concentration *in vitro*. We have also shown here that IHC on the LuCaP 86.2 xenograft that predominantly expresses one of the AR C-terminal truncated splice variants had a similar staining pattern to 24% of the metastatic CRPC tissues (Figure 1 and Figure 3). Since the constitutively active AR variants could translocate to the nucleus without ligand, they would account for the different staining results in the nucleus using 2 AR antibodies. However, it should also be noted that the decreased immunoreactivity in the nucleus with C-terminal AR antibody is not an all or none phenomenon, and that a percentage of C-terminal AR staining is also found in the nucleus of these tissues. This would not be unexpected since we have shown that AR^v567es^ dimerizes with AR^FL^ and can cause translocation of the AR^FL^ into the nucleus in the absence of ligand [25].

One of our limitations was the inability of the assay to separate constitutively active from inactive C-terminal deleted AR proteins. To address this, we examined the expression of known AR regulated proteins. We observed a decrease in PSA and PSMA expression in the CRPC sites with the loss of C-terminal AR staining. However the decrease in PSA and PSMA expression was limited, suggesting constitutively active AR variants constitute a significant portion of the C-terminal truncated AR variants in CRPC.

In order to further explore the activity of AR variants among the tissues, we selected a group of genes that are purported to be AR variant regulated in human tissue [26], [29]. The expression levels of these genes were determined by quantitative RT-PCR in subsets of tissues where cDNA was available. The variant-associated genes that were elevated in the N+C↓ tissues compared to N+C+ were cell cycle genes associated with transition through G2-M of the cell cycle or glucuronidation of androgens, UGT2B17. Of interest, UGT2B17 is two logs higher in the LuCaP 86.2 line compared to LuCaP35. We have previously shown that LuCaP 86.2 has very low levels of intratumoral steroid and high AR^v567es^ regardless of whether the host is castrated or not [25]. In contrast LuCaP 35 does not express AR splice variants unless castrated and even then requires an inhibitor of androgen synthesis e.g. abiraterone to express AR^v467es^ [25], [38]. These data suggest that not only do constitutively active AR splice variants increase AR- regulated cell cycle genes, but by increasing UGT2B17 and decreasing intracellular steroid, they further propagate an intracellular milieu that favors further AR-variant synthesis.

We observed considerable heterogeneity in AR staining within and between patients with CRPC. This suggests that the truncation and/or loss of the AR are not necessarily early clonal events in the development of PCa, rather late stage events occurring independently of one another in CRPC. As proposed by us and others, C-terminal AR loss, may be associated with mutations, chromosomal rearrangements, or splicing events that occur after androgen withdrawal [23], [25]. Our results suggest that a large proportion of the variants with a C-terminal loss are constitutively active. Therefore, the mechanisms involved in the acquisition of AR variant phenotype and the number of variants that are constitutively active in CRPC will require further investigation.

An important question for a patient in whom there is a conversion to decreased C-terminal AR is whether or not it should effect treatment decisions, especially since two new treatments for PCa that has recurred following “traditional” ADT, i.e. abiraterone and MDV-3100, require the LBD of the AR to be effective [26], [28]. We have shown that constitutively active AR splice variants form heterodimers with the AR^FL^ and enhance AR^FL^ transactivation by ligand [25]. Furthermore, Watson and colleagues have subsequently shown that AR variants may require AR^FL^ for activity and thus respond to MDV-3100 [28]. Here, we show that AR^v567es^ is increased after androgen withdrawal and androgen receptor inhibitor MVD-3100 treatment, which correlates with our findings herein of an increase in AR variants with a decrease in C-terminal IHC reactivity in CRPC. However, as shown herein, there is heterogeneity among the metastatic sites with regard to variant status and this confounds potential clinical management decisions. Further studies are required to determine whether assessments of AR splice variants will be useful in stratifying patients for AR pathway-targeted therapies.

Hornberg et al published an independent study showing increased mRNA levels of 3 specific AR splice variants (AR-V1, AR-V7 and AR^v567es^) in CRPC [29], which is consistent with our findings. However, there are several important differences between these two studies. First, our approach is able to detect the distribution of all C-terminal truncated AR variant proteins in the paraffin embedded tissue. Although AR-V1, AR-V7 and AR^v567es^ are the three variants identified in human tissue so far, emerging studies have suggested the existence of other AR variants which should also be considered in regard to evaluation of total AR functions. Our study showed a relatively complete profile of C-terminal truncated AR variants in CRPC patients, and found AR regulated gene products are associated with the distribution of AR variants. Secondly, we investigated multiple metastatic sites for each CRPC patient, whereas only single metastatic site per patient was assessed in Hornberg's study. Third, in our study, we did not find any correlation between AR variants and time from diagnosis to death or starting androgen ablation treatment to death. This would not be unexpected since in Hornberg's study, the AR variants were detected at a specific event prior to death, i.e. pathologic fracture; whereas our tissues were collected at the time of death and thus the time from appearance of variants to death is unknown. Furthermore, we have recently shown that recurrence of human PCa xenografts following castration and inhibition of steroid synthesis with the CYP17 inhibitor abiraterone may be associated with either AR splice variants expression or intracrine steroidogenesis [38]. As we pointed out earlier, the expression of AR variants between metastatic sites is heterogeneous and is a very common event seen within and between CRPC patients. This should be taken into consideration when determining if an AR variant(s) detected in a single metastatic site should affect clinical treatment decisions.
